# Emergency imaging protocols for pregnant patients: a multi-institutional and multi- specialty comparison of physician education

**DOI:** 10.1007/s10140-024-02284-4

**Published:** 2024-10-14

**Authors:** Liesl Eibschutz, Max Yang Lu, Payam Jannatdoust, Angela C. Judd, Claire A. Justin, Brandon K.K. Fields, Natalie L. Demirjian, Madan Rehani, Sravanthi Reddy, Ali Gholamrezanezhad

**Affiliations:** 1 Department of Internal Medicine, University of Virginia Hospital, Charlottesville, VA USA; 2 Division of Emergency Radiology, Los Angeles General Hospital, Los Angeles, CA 90033 USA; 3 Tehran University of Medical Sciences, Tehran, Iran; 4 Department of Obstetrics and Gynecology, Icahn School of Medicine at Mount Sinai/South Nassau, New York, NY USA; 5 Department of Pediatrics, Stanford University, Palo Alto, CA USA; 6 Department of Radiology & Biomedical Imaging, University of California, San Francisco, CA USA; 7 College of Medicine – Tucson, University of Arizona, Tucson, AZ USA; 8 Department of Radiology, Massachusetts General Hospital, Cambridge, MA USA; 9 Department of Medicine, Yale School of Medicine, New Haven, CT USA

**Keywords:** Imaging in pregnancy, Ionizing radiation in pregnancy, Acute trauma in pregnancy, Appendicitis in pregnant patients, Pulmonary embolism in pregnant patients

## Abstract

**Purpose:**

Previous studies have demonstrated that radiologists and other providers perceive the teratogenic risks of radiologic imaging to be higher than they actually are. Thus, pregnant patients were less likely to receive ionizing radiation procedures. While it is imperative to minimize fetal radiation exposure, clinicians must remember that diagnostic studies should not be avoided due to fear of radiation, particularly if the imaging study can significantly impact patient care. Although guidelines do exist regarding how best to image pregnant patients, many providers are unaware of these guidelines and thus lack confidence when making imaging decisions for pregnant patients. This study aimed to gather information about current education, confidence in, and knowledge about emergency imaging of pregnant women among radiology, emergency medicine, and OB/GYN providers.

**Methods:**

We created and distributed an anonymous survey to radiology, emergency medicine, and OB/GYN providers to evaluate their knowledge and confidence in imaging pregnant patients in the emergent setting. This study included a questionnaire with the intent of knowing the correct answers among physicians primarily across the United States (along with some international participation). We conducted subgroup analyses, comparing variables by specialty, radiology subspecialty, and training levels. Based on the survey results, we subsequently developed educational training videos.

**Results:**

108 radiologists, of which 32 self-identified as emergency radiologists, ten emergency medicine providers and six OB/GYN clinicians completed the survey. The overall correct response rate was 68.5%, though performance across questions was highly variable. Within our 18-question survey, four questions had a correct response rate under 50%, while five questions had correct response rates over 90%. Most responding physicians identified themselves as either “fairly” (58/124, 47%) or “very” (51/124, 41%) confident. Amongst specialties, there were differences in performance concerning the knowledge assessment (*p* = 0.049), with the strongest performance from radiologists. There were no differences in knowledge by training level (*p* = 0.4), though confidence levels differed significantly between attending physicians and trainees (*p* < 0.001).

**Conclusion:**

This study highlights deficiencies in knowledge to support appropriate decision-making surrounding the imaging of pregnant patients. Our results indicate the need for improved physician education and dissemination of standardized clinical guidelines.

## Introduction

Amongst pregnant women, various conditions warrant the use of medical imaging in emergent settings, such as deep vein thrombosis, pulmonary embolism, acute appendicitis, cerebral venous sinus thrombosis, acute cholecystitis, and trauma [1–3]. However, given the teratogenic risks of radiation exposure, special considerations must be taken into account when imaging pregnant patients [4]. As there is no universally agreed upon threshold for radiation exposure during pregnancy, many authors argue that there is no safe level [5]. While ultrasound (US) and magnetic resonance imaging (MRI) do not involve ionizing radiation, other commonly employed techniques such as radiography or X-ray, computerized tomography (CT), fluoroscopic examinations, and positron emission tomography (PET) do. Thus, in emergencies involving pregnant or potentially pregnant patients, clinicians must act urgently while considering many complex factors, such as the safety and relevance of the study, concerns regarding radiation dose, and the most appropriate imaging modality for both the patient and fetus.

Physicians involved in imaging pregnant patients in emergent settings include emergency medicine (EM), obstetrics and gynecology (OB/GYN), and radiology. Emergency physicians are often consulted for pregnant patients with chest pain and severe trauma, whereas possible pregnancy-related abdominal pain and minor trauma in the late 2nd or 3rd trimester are most likely to be seen in obstetrics triage [6, 7]. Radiologists are responsible for obtaining the studies and reporting back to the ordering physician. Thus, each role is directly involved in caring for pregnant patients, and each must know the risks, benefits, and imaging protocols.

While radiologic imaging plays a beneficial role in investigating many conditions in pregnancy, it also has potential to cause harm [8–10]. Yet, radiation doses associated with these imaging modalities are typically much lower than the levels yielding fetal harm, and thus, these techniques should not be withheld if the benefit outweighs the risk. While the American College of Radiology (ACR) and the American College of Obstetricians and Gynecologists (ACOG) have published practice guidelines [5, 11], their extent of use has not been widely studied. In addition to guidelines of professional societies, several publications provide useful resources such as those published by the International Atomic Energy Agency (IAEA) and the International Commission on Radiological Protection (ICRP) Publication 84 on pregnancy and medical radiation [12, 13]. In this study, we aimed to gather information about current education, knowledge and confidence about emergency imaging of pregnant women among providers of varying specialty training backgrounds.

## Methods

### Participants and data collection

Our study involved an initial data-gathering phase followed by an educational intervention. In the first phase, we created and distributed an anonymous, self-administered, cross-sectional survey to radiology, EM, and OB/GYN residents, fellows, and attendings. The survey was sent to medical resident associations and other medical associations across the United States, as well as physicians in some international countries (Hungary, India, Slovenia, Italy, Saudi Arabia, Croatia, Pakistan, and Canada). Participants self-identified their background information and 70.2% (87/124) of respondents reported being from the United States. 17.7% (22/124) did not report a country of residence, 5.6% (7/124) were from Canada, 3.2% (were from Hungary and 0.8% (1/124) of respondents were from India, Italy, Saudi Arabia, and Slovenia each. This survey was created via REDCap ^TM^ (Research Electronic Data Capture), a HIPAA-compliant software used for the secure collection of survey-based data. The survey results then were utilized to create eight educational segments that covered a wide range of topics based on where the survey indicated weaknesses. The project was approved by the University of Southern California Institutional Review Board and was made possible with the support of the American Society of Emergency Radiology and the Kathirkamanathan Shanmuganathan Research Grant.

### Questionnaire

The first part of the survey included demographic information such as the participant’s gender, specialty, level of training, and physicians’ knowledge sources drawn upon for decisions regarding imaging of pregnant patients. It also included questions about the clinician decision-making frequency in imaging pregnant patients and their confidence in doing so, which were assessed via Likert Scales. The second part of the survey included 18 questions assessing clinician knowledge adapted from practice parameters published by the American College of Radiology (ACR), the Society for Pediatric Radiology (SPR), the IAEA, and the ICRP [12]. Questions were primarily multiple-choice questions with just one correct answer out of 5–7 options presented. Additionally, the survey included two true/false or yes/no questions and one where participants were asked to select all applicable answers out of 11 options. A detailed explanation and the reference sources were provided after recipients answered each question.

The 18 questions in the knowledge assessment part of the survey were initially segmented into two categories: general theoretical knowledge and clinical decision-making. These categories distinguished between questions requiring a foundational understanding of radiological imaging’s effects and safety during pregnancy and those necessitating practical decision-making in clinical scenarios. The clinical decision-making category was further divided into four subcategories: gastrointestinal (GI) imaging, neuroradiology, trauma imaging, and cardiopulmonary imaging. Notably, one question within the cardiopulmonary imaging subcategory (question 7) originated from the general theoretical knowledge group, as it pertained theoretically to a cardiopulmonary scenario. Table 1 delineates each question, its correct response, and its classification in detail.

**Table 1:** The general theoretical knowledge and clinical decision-making questions, classification, correct answers, and all possible answer choices in the knowledge assessment part of the survey

Question number	Question	Correct answer	Subdomain	All answer options
Q1	At what gestational age is the growing fetus most susceptible to diminished IQ from radiation?	B. 8-15 weeks post conception	General theoretical knowledge	(A) 0-7 weeks post conception **(B) 8-15 weeks post conception** (C) 16-25 weeks post conception (D) 26-33 weeks post conception (E) >34 weeks post conception
Q2	What is the most likely result when the embryo is exposed to radiation under 100 mGy within the first two weeks post conception?	E. No effect	General theoretical knowledge	(A) Malformation (B) Fetal Death (C) Growth Retardation (D) Cataracts **(E) No effect**
Q3	Which 5 diagnostic tests do not require verification of pregnancy status according to the American College of Radiology? Please select 5	A. Chest or extremity Radiograph C. Chest CT D. Head CT E. MRI Brain and >H. Mammography	Clinical decision making	**(A) Chest or extremity Radiograph** (B) CT abdomen/ pelvis **(C) Chest CT (D) Head CT (E) MRI Brain** (F) PET/CT (G) Hysterosalpingography **(H) Mammography** (I) Pelvic Angiography (J) 99mTc-MDP Bone Scan
Q4	What is the most likely outcome when a 19-week post conception fetus is exposed to a diagnostic dose of radiation (assume < 30 mGy)?	>F. No effect	General theoretical knowledge	(A) Induced cancer (B) Fetal loss (C) Congenital malformations (D) Fetal growth restrictions (E) Developmental delay **(F) No effect**
Q5	Evaluate the accuracy of the statement: “Radiation doses resulting from most diagnostic procedures present no substantial risk of causing fetal death, malformation, or impairment of mental development”	A. True	General theoretical knowledge	**(A) True** (B) False
Q6	A 28-year-old, 19-week pregnant woman presents to the ED with presumed pulmonary embolism. An initial chest radiograph is abnormal. What test would be most definitive for diagnosing PE?	E. CT pulmonary angiogram	Clinical decision making, Cardiopulmonary imaging	(A) Duplex Ultrasound LE (B) MRI without contrast (C) MRI with contrast (D) D-Dimer **(E) CT pulmonary angiogram** (F) V/Q scintigraphy
Q7	Your colleague hears you are working up PE in a pregnant patient and asks you how the radiation exposure for both mother and fetus differs between the following two tests: CT pulmonary angiogram and V/Q scintigraphy. Which most accurately describes the radiation exposure in these two tests? (Assume dose-modulation techniques have NOT been utilized)	E. V/Q scintigraphy exposes the fetus to more radiation but exposes the mother to less radiation than CTPA	General theoretical knowledge, Cardiopulmonary imaging	(A) CTPA exposes both the fetus and the mother to more radiation than a V/Q scintigraphy (B) V/Q scintigraphy exposes both the fetus and the mother to more radiation than a CTPA (C) The radiation exposure to the fetus is roughly the same between the two modalities but V/Q scintigraphy exposes the mother to more radiation (D) The radiation exposure to the fetus is roughly the same between the two modalities but CTPA exposes the mother to more radiation **(E) V/Q scintigraphy exposes the fetus to more radiation but exposes the mother to less radiation than CTPA** (F) CTPA exposes the fetus to more radiation but exposes the mother to less radiation than V/Q scintigraphy
Q8	A 35-year-old woman, 34 weeks pregnant, is brought to the ED after a motor vehicle accident and complains of diffuse abdominal pain. Focused abdominal ultrasonography for trauma (FAST) shows no free intraperitoneal fluid. Which of the following is the most appropriate next step	A. Abdominal/Pelvic CT scan	Clinical decision making, Trauma imaging	**(A) Abdominal/Pelvic CT scan.** (B) MRI with contrast (C) MRI without contrast (D) Diagnostic peritoneal lavage. (E) Exploratory laparotomy. (F) Pelvic angiography
Q9	Now assume that our patient with a negative FAST is in a resource-poor setting, or your facility’s CT scanner is down. She is also hemodynamically unstable What would be the most appropriate next step?	C. Diagnostic peritoneal lavage	Clinical decision making, Trauma imaging	(A) Abdominal Radiograph (B) Pelvic ultrasound **(C) Diagnostic peritoneal lavage** (D) Exploratory laparotomy (E) Pelvic angiography
Q10	A 27-year-old woman in her 10th week of pregnancy and a history of hypertension presents to the ED with left sided weakness in her face and arm. Which first diagnostic test would be most appropriate for this patient?	C. Head CT without contrast	Clinical decision making, Neuroradiology	(A) Carotid duplex Ultrasound (B) Head CT with contrast **(C) Head CT without contrast** (D) MRI with contrast (E) MRI without contrast (F) Transthoracic echocardiography
Q11	A 27-year-old patient in her third trimester presents with suspected ankle fracture after falling down the stairs. Is an x-ray appropriate to evaluate this patient’s suspected fracture?	A. Yes, medically indicated radiologic examinations remote from the fetus (e.g., radiographs of the chest or extremities) can be safely done at any time during pregnancy	Clinical decision making, Trauma imaging	**(A) Yes**,** medically indicated radiologic examinations remote from the fetus (e.g.**,** radiographs of the chest or extremities) can be safely done at any time during pregnancy** (B) No, radiation from diagnostic studies may result in harmful effects on the child and should be avoided
Q12	A 35-year-old patient in her 22nd week of pregnancy and a history of rheumatic heart disease presents with sensations of a rapid, fluttering heartbeat and pitting edema in her lower extremities.Which of the following would be the best imaging technique when examining the patient?	D. Echocardiogram	Clinical decision making, Cardiopulmonary imaging	(A) Chest CT (B) Cardiac MRI with contrast (C) Cardiac MRI without contrast **(D) Echocardiogram** (E) Transesophageal echocardiography (TEE)
Q13	The following prompt pertains to both questions 13 and 14: A 22-week pregnant patient arrives to the ED with acute abdominal pain that is localized to her right lower quadrant. What is the first diagnostic modality to rule out appendicitis in this patient?	A. Ultrasound	Clinical decision making, GI imaging	**(A) Ultrasound** (B) CT with IV contrast (A) CT without IV contrast (B) MRI with contrast (C) MRI without IV contrast
Q14	The following prompt pertains to both questions 13 and 14: A 22-week pregnant patient arrives to the ED with acute abdominal pain that is localized to her right lower quadrant. The imaging modality chosen in Question 13 was unable to visualize the appendix. What is the next best diagnostic modality to rule out appendicitis in this patient?	E. MRI without contrast	Clinical decision making, GI imaging	(A) Ultrasound (B) CT with IV contrast (C) CT without IV contrast (D) MRI with contrast **(E) MRI without IV contrast**
Q15	The following prompt pertains to both questions 15 and 16: Assume a patient in her 1st trimester presents with choledocholithiasis. Which of the following is the most sensitive imaging modality for detecting common bile duct stones in a 1st trimester pregnant patient?	C. Endoscopic Ultrasound	Clinical decision making, GI imaging	(A) Plain Radiograph (B) Transabdominal Ultrasound **(C) Endoscopic Ultrasound** (D) CT abdomen (E) Magnetic resonance cholangiopancreatography (MRCP) D. HIDA scan E. Endoscopic retrograde cholangiopancreatography (ERCP)
Q16	Now assume that this patient is in her 2nd trimester and transabdominal ultrasound imaging was nondiagnostic. Which of the following is the best imaging modality for detecting common bile duct stones in a 2nd trimester pregnant patient?	E. Magnetic resonance cholangiopancreatography (MRCP)	Clinical decision making, GI imaging	(A) Plain Radiograph (B) Transvaginal Ultrasound (C) Endoscopic Ultrasound (D) CT abdomen **(E) Magnetic resonance cholangiopancreatography (MRCP)** (F) HIDA scan (G) Endoscopic retrograde cholangiopancreatography (ERCP)
Q17	The following prompt pertains to both questions 17 and 18: A 23-year-old pregnant woman arrives to the emergency room stating she had a sudden onset of the “worst headache of my life”. Which imaging modality would be the first choice for the suspected diagnosis?	B. CT brain without contrast	Clinical decision making, Neuroradiology	(A) CT brain with contrast **(B) CT brain without contrast** (C) CT angiography (D) CT Venography (E) MRI brain (F) MR angiography
Q18	Now which two imaging modalities could be used next to localize the underlying pathology confirmed by the imaging technique used in Question 17? Please select 2 choices.	C. CT angiography and F. MR angiography	Clinical decision making, Neuroradiology	(A) CT brain with contrast (B) CT brain without contrast **(C) CT angiography** (D) CT Venography (E) MRI brain **(F) MR angiography**

### Educational intervention

An hour-long educational video was created with eight different segments based on the results of the survey. These segments delineated the basics of how to image pregnant patients with gastrointestinal, obstetrics and gynecological, neurologic, and orthopedic conditions, blunt and penetrating trauma, and deep vein thrombosis and pulmonary embolism. Non-clinical topics such as radiation dosing, risks of radiation exposure, and imaging appropriateness and safety were also discussed. These topics were considered the most common clinical scenarios experienced by pregnant patients. This educational intervention aimed to help clinicians feel more comfortable treating pregnant patients presenting to the emergency department. The acknowledgments section contains a link to the educational video and a complete breakdown of each segment’s duration.

### Statistical analyses

For analyses of demographic and baseline data, as well as the frequency of decision and confidence assessed via the Likert scale, Fisher’s exact test was used for categorical data, and the Kruskal-Wallis rank sum test was used to compare continuous variables. The assessment scores were calculated as the percentage of correct responses across all questions and within each subdomain. No score was awarded for partially correct answers. The percentages of correct responses were compared across specialty and training level subgroups using non-parametric Kruskal-Wallis rank sum tests. Significant differences between subgroups prompted post-hoc pairwise comparisons, which were conducted via Dunn’s test with false discovery rate (FDR) correction using the Benjamini-Hochberg method. Due to the non-parametric distribution of continuous variables, median and interquartile range (IQR) were utilized for expression. All statistical analyses were conducted using R statistical software version 4.2.1 and Python version 3.11 for data visualization, incorporating “gtsummary” [14], “ggalluvial ” [15], “webr,” “Scipy,” “Seaborn,” and “statannotations ” packages [16]. *p* or *q* < 0.05 was considered statistically significant.

## Results

### Sample characteristics

**Fig. 1:**
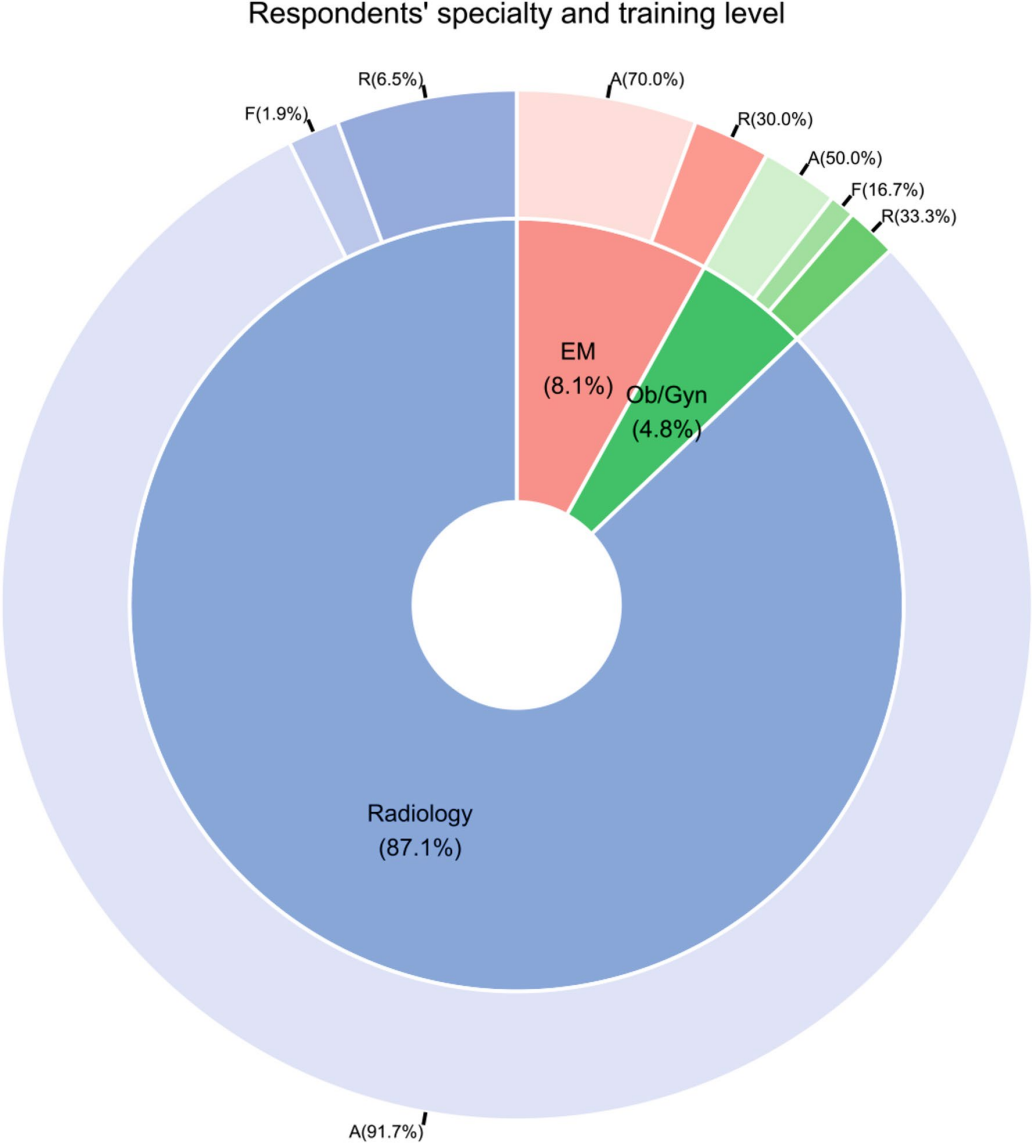
Specialty and training level of respondents. **A**: Attending physician; EM: Emergency medicine; F: Fellowship; R: Resident physician; OB/GYN: Obstetrics and gynecology

**Fig. 2:**
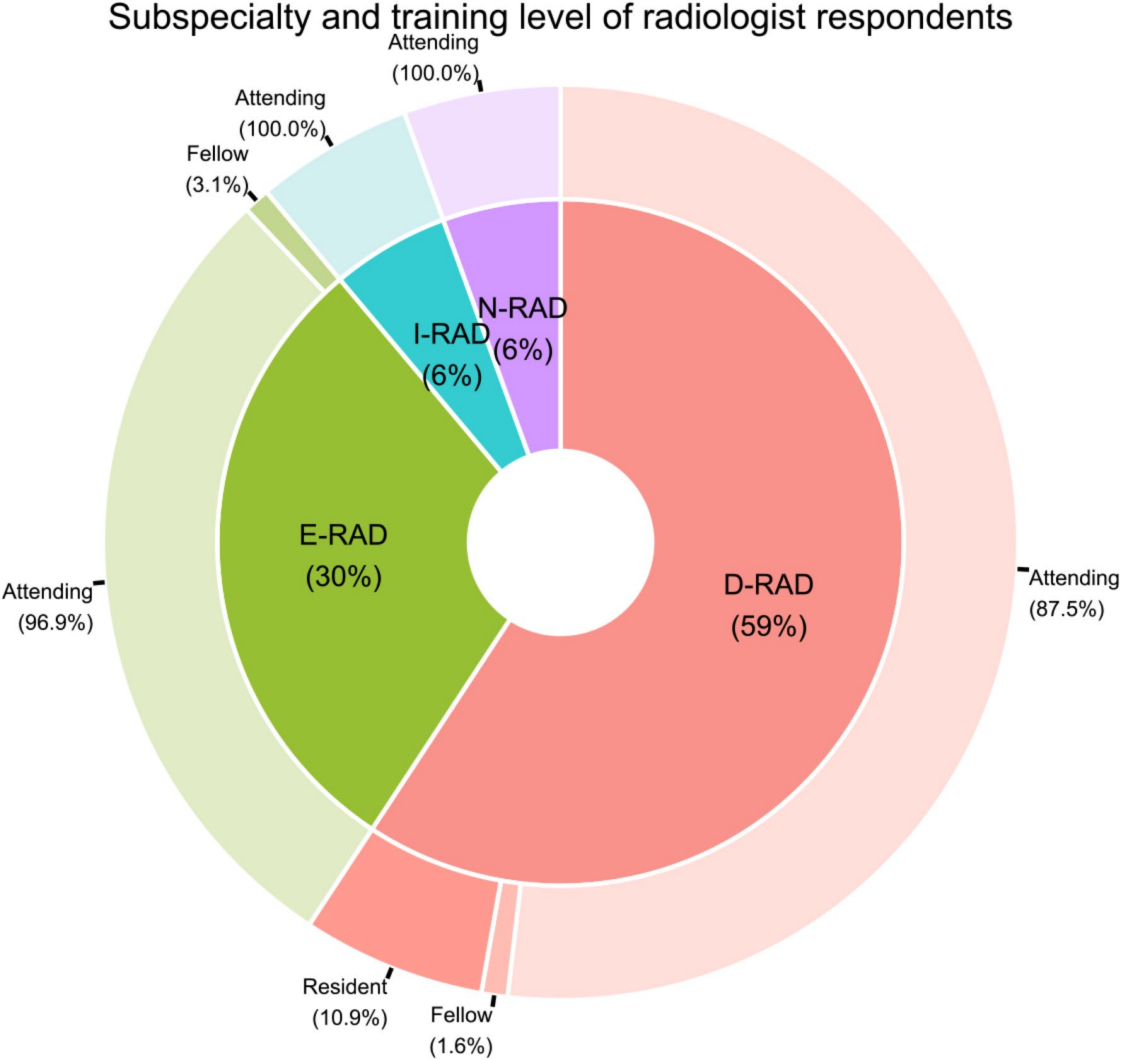
Subspecialty and training level of radiologist respondents. D-RAD: Diagnostic radiology; E-RAD: Emergency radiology; I-RAD: Interventional radiology; N-RAD: Neuroradiology

From the initial pool of potential participants who received the survey (*n* = 812), 223 individuals responded, with 124 remaining after post-eligibility and completeness assessment, resulting in a response rate of 15.3%. The criteria utilized to determine eligibility was based on whether the respondent completed the entire survey or not. Surveys were eliminated from consideration if the participant did not adequately answer all questions. A substantial majority of these respondents (108/124, 87.1%) identified as practicing radiologists or radiology trainees, succeeded by EM physicians (10/124, 8.1%) and OB/GYN specialists (6/124, 4.8%), with attending physicians predominating within each group (Fig. 1). The subspecialty and training status of radiologist respondents, detailed in Fig. 2, show a majority in the field of diagnostic radiology (64/108, 59%), followed by emergency radiology (32/108, 30%), interventional radiology (6/108, 6%), and neuroradiology (6/108, 6%). Additional demographic and general information are provided in Table 2.

**Table 2:** Characteristics of respondents

		Specialty			
Characteristic	Overall, *N* = 124^a^	EM, *N* = 10^a^	Ob/Gyn, *N* = 6^a^	Radiology, *N* = 108^a^	*p*-value^b^
**Gender**					0.8
Female	35% (41/118)	30% (3/10)	50% (3/6)	34% (35/102)	
Male	65% (77/118)	70% (7/10)	50% (3/6)	66% (67/102)	
Non-binary / prefers not to tell	6	0	0	6	
**Training level**					**0.006**
Attending	88% (109/124)	70% (7/10)	50% (3/6)	92% (99/108)	
Fellowship	2.4% (3/124)	0% (0/10)	17% (1/6)	1.9% (2/108)	
Residency	9.7% (12/124)	30% (3/10)	33% (2/6)	6.5% (7/108)	
**Frequency of decision for pregnant patients**					**< 0.001**
1.Never	1.6% (2/124)	0% (0/10)	0% (0/6)	1.9% (2/108)	
2.Rarely (< 1 per week)	49% (61/124)	0% (0/10)	0% (0/6)	56% (61/108)	
3.Sometimes (> 1 per week)	31% (38/124)	70% (7/10)	50% (3/6)	26% (28/108)	
4.Often (> 1 per day)	12% (15/124)	30% (3/10)	33% (2/6)	9.3% (10/108)	
5.Always (> 1 per shift)	6.5% (8/124)	0% (0/10)	17% (1/6)	6.5% (7/108)	
**Likert scale for frequency (1–5)**	2.0 [2.0–3.0]	3.0 [3.0–3.8]	3.5 [3.0–4.0]	2.0 [2.0–3.0]	**< 0.001**
**Confidence level**					0.6
1.Not at all	1.6% (2/124)	0% (0/10)	0% (0/6)	1.9% (2/108)	
2.Slightly	10% (13/124)	0% (0/10)	17% (1/6)	11% (12/108)	
3.Fairly	47% (58/124)	70% (7/10)	33% (2/6)	45% (49/108)	
4.Very	41% (51/124)	30% (3/10)	50% (3/6)	42% (45/108)	
**Likert scale for confidence (1–4)**	3.0 [3.0–4.0]	3.0 [3.0–3.8]	3.5 [3.0–4.0]	3.0 [3.0–4.0]	> 0.9
**Knowledge Source**					**0.020**
ACR	48% (60/124)	30% (3/10)	0% (0/6)	53% (57/108)	
Asking attending physician/ colleagues	18% (22/124)	30% (3/10)	50% (3/6)	15% (16/108)	
Other sources	34% (42/124)	40% (4/10)	50% (3/6)	32% (35/108)	

a: % (n/N); Median [IQR]

b: Fisher’s exact test; Kruskal-Wallis rank sum test

ACR: American college of radiology; EM: Emergency medicine; IQR: Inter-quartile range; Ob/Gyn: Obstetrics and gynecology

### Decision-making according to specialty

Notably, significant differences were observed in the frequency of decision-making for pregnant patients among the three subgroups (*p* < 0.001), with all responding OB/GYN and EM clinicians reporting weekly decision-making, compared to 57.9% (63/108) of radiologists who reported less frequent decision-making (Table 2). Overall, physicians reported a median frequency of “rarely” (less than once per week) making imaging decisions for pregnant patients. Just 1.6% (2/124) of respondents reported they never engaged in pregnant patient imaging decisions, and 18.5% (23/124) of respondents reported engaging in such decisions with a frequency of more than once per day or more. In making judgments on this topic, physicians most often described themselves as “fairly” (58/124, 47%) or “very” (51/124, 41%) confident, with no differences between specialties. Overall, amongst all physicians surveyed, regarding the knowledge sources consulted in deciding how best to image pregnant patients, the ACR guidelines were the preferred source (60/124, 48%), though asking attending physicians/colleagues (22/124, 18%) was a common practice as well. There were significant differences between specialties in terms of knowledge sources drawn upon (*p* = 0.020). The ACR guidelines were preferred by 53% (57/108) of radiologists, 30% (3/10) of EM physicians, but none (0/6) of the OB/GYN physicians surveyed.

### Decision-making according to training level

Table 3 further stratifies the data by training level, highlighting an unsurprising significant disparity in confidence levels between trainees and attending physicians (*p* < 0.001), with a higher proportion of attending physicians expressing confidence in imaging-related decisions in pregnant patients. No statistically significant differences were reported regarding the frequency of imaging decisions between attending physicians and trainees (*p* = 0.8), though the knowledge sources on which physicians and trainees drew were significantly different (*p* = 0.004). These statistics were not significant when compared across different radiology subspecialties.

**Table 3:** Characteristics of respondents, stratified by training level

		Training level			
Characteristic	**Overall**, *N* = 124^a^	Attending, *N* = 109^a^	Fellowship, *N* = 3^a^	Residency, *N* = 12^a^	*p*-value^b^
**Gender**					0.9
Female	35% (41/118)	34% (35/103)	33% (1/3)	42% (5/12)	
Male	65% (77/118)	66% (68/103)	67% (2/3)	58% (7/12)	
Non-binary - prefers not to tell	6	6	0	0	
**Training year**					NA
1	-	-	0% (0/3)	33% (4/12)	
2	-	-	100% (3/3)	17% (2/12)	
3	-	-	-	25% (3/12)	
4	-	-	-	25% (3/12)	
**Specialty**					**0.006**
EM	8.1% (10/124)	6.4% (7/109)	0% (0/3)	25% (3/12)	
Ob/Gyn	4.8% (6/124)	2.8% (3/109)	33% (1/3)	17% (2/12)	
Radiology	87% (108/124)	91% (99/109)	67% (2/3)	58% (7/12)	
**Frequency of decision for pregnant patients**					> 0.9
1.Never	1.6% (2/124)	1.8% (2/109)	0% (0/3)	0% (0/12)	
2.Rarely (< 1 per week)	49% (61/124)	49% (53/109)	67% (2/3)	50% (6/12)	
3.Sometimes (> 1 per week)	31% (38/124)	30% (33/109)	33% (1/3)	33% (4/12)	
4.Often (> 1 per day)	12% (15/124)	13% (14/109)	0% (0/3)	8.3% (1/12)	
5.Always (> 1 per shift)	6.5% (8/124)	6.4% (7/109)	0% (0/3)	8.3% (1/12)	
**Likert scale for frequency (1–5)**	2.0 [2.0–3.0]	2.0 [2.0–3.0]	2.0 [2.0–2.5]	2.5 [2.0–3.0]	0.8
**Confidence level**					**< 0.001**
1.Not at all	1.6% (2/124)	0% (0/109)	0% (0/3)	17% (2/12)	
2.Slightly	10% (13/124)	8.3% (9/109)	33% (1/3)	25% (3/12)	
3.Fairly	47% (58/124)	46% (50/109)	33% (1/3)	58% (7/12)	
4.Very	41% (51/124)	46% (50/109)	33% (1/3)	0% (0/12)	
**Likert scale for confidence (1–4)**	3.0 [3.0–4.0]	3.0 [3.0–4.0]	3.0 [2.5–3.5]	3.0 [2.0–3.0]	**< 0.001**
**Knowledge Source**					**0.004**
ACR	48% (60/124)	51% (56/109)	33% (1/3)	25% (3/12)	
Asking attending physician/ colleagues	18% (22/124)	14% (15/109)	0% (0/3)	58% (7/12)	
Other sources	34% (42/124)	35% (38/109)	67% (2/3)	17% (2/12)	

a: % (n/N); Median [IQR]

b: Fisher’s exact test; Kruskal-Wallis rank sum test

ACR: American college of radiology; EM: Emergency medicine; IQR: Inter-quartile range; Ob/Gyn: Obstetrics and gynecology

### General test performance

**Fig. 3:**
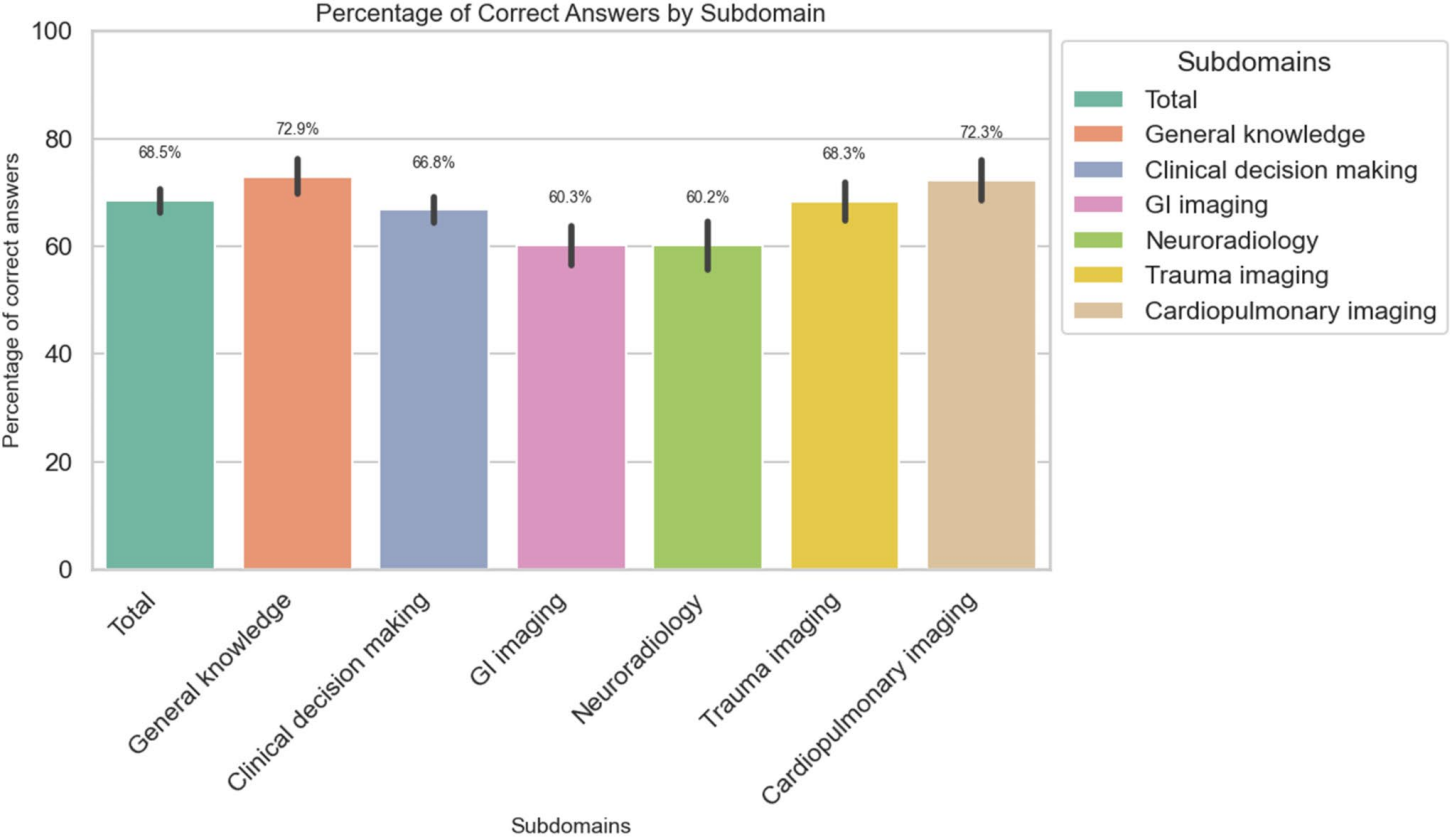
Bar plots representing the percentage of correct answers in each subdomain and error bars representing 95% confidence intervals. GI: Gastrointestinal

**Fig. 4:**
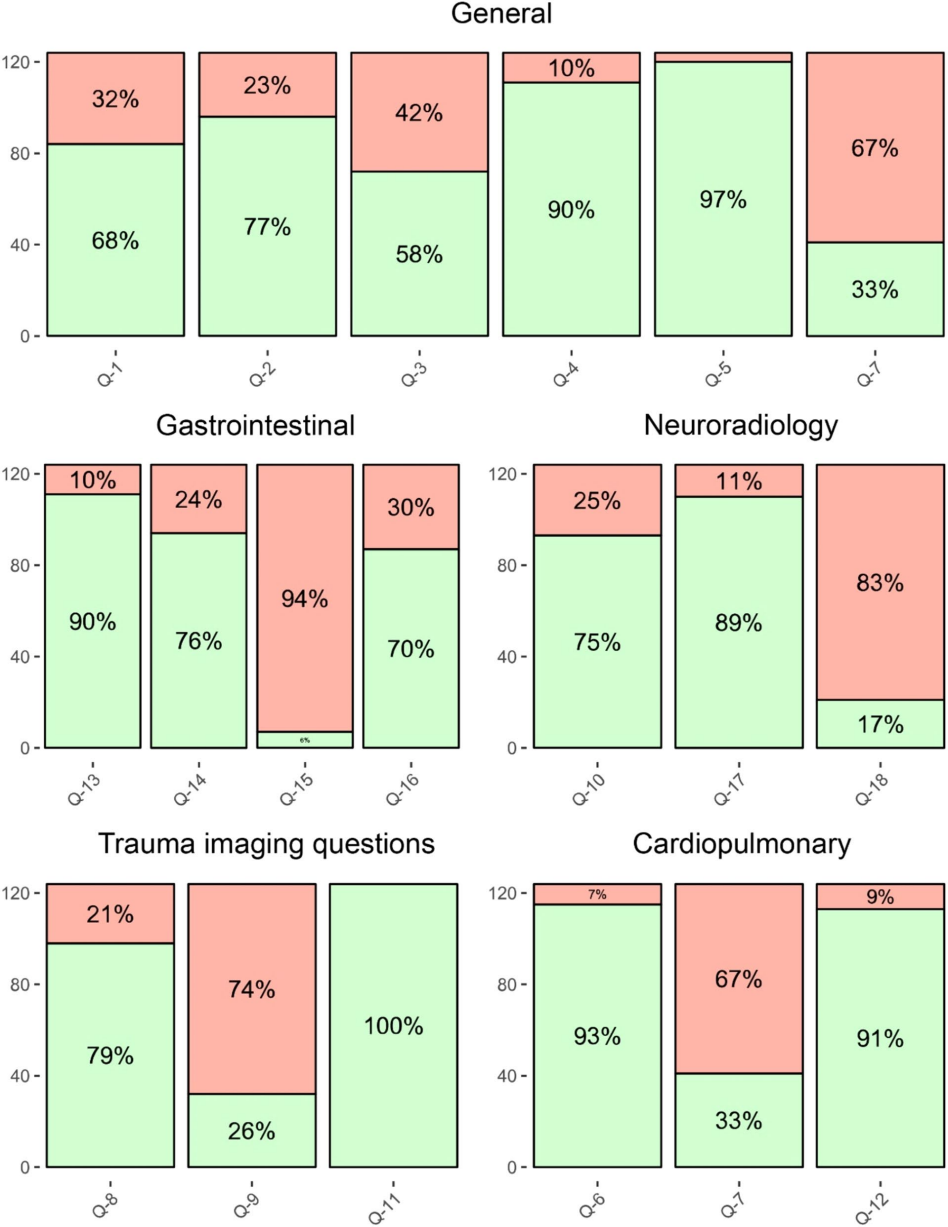
Diagram of the percentage of respondents with correct (green) and incorrect (red) answers to each question. Furthermore, question seven is present both in cardiopulmonary imaging and general knowledge because both cardiopulmonary and general knowledge were required to answer it

The overall correct response rate was 68.5%, with neuroradiology and gastrointestinal imaging scenarios having the lowest correct answer rates (60.2% and 60.3%, respectively) and the general knowledge and cardiopulmonary imaging domains having the highest correct answer rates (72.9% and 72.3% respectively) (Fig. 3). Figure 4 showcases a diagram of the percentage of respondents with correct and incorrect answers. Detailed analysis of question-specific correct response rates identified questions 7, 9, 15, and 18 as the most challenging, with less than 50% correct responses (Table 4).

**Table 4:** Statistics of the questions with the lowest correct answer rate (questions 7,9,15, and 18). There was no significant difference in performance between radiologists and non-radiologists

Characteristic	Overall, *N* = 124^a^	Non-radiology, *N* = 16^a^	Radiology, *N* = 108^a^	*p*-value^b^	q-value^c^
Question 7	33% (41/124)	19% (3/16)	35% (38/108)	0.2	0.5
Question 9	26% (32/124)	25% (4/16)	26% (28/108)	> 0.9	> 0.9
Question 15	5.6% (7/124)	6.3% (1/16)	5.6% (6/108)	> 0.9	> 0.9
Question 18	17% (21/124)	0% (0/16)	19% (21/108)	0.071	0.3

a: (n/N)

b: Pearson’s Chi-squared test; Fisher’s exact test

c: False discovery rate correction for multiple testing

### Test performance according to specialty

**Fig. 5:**
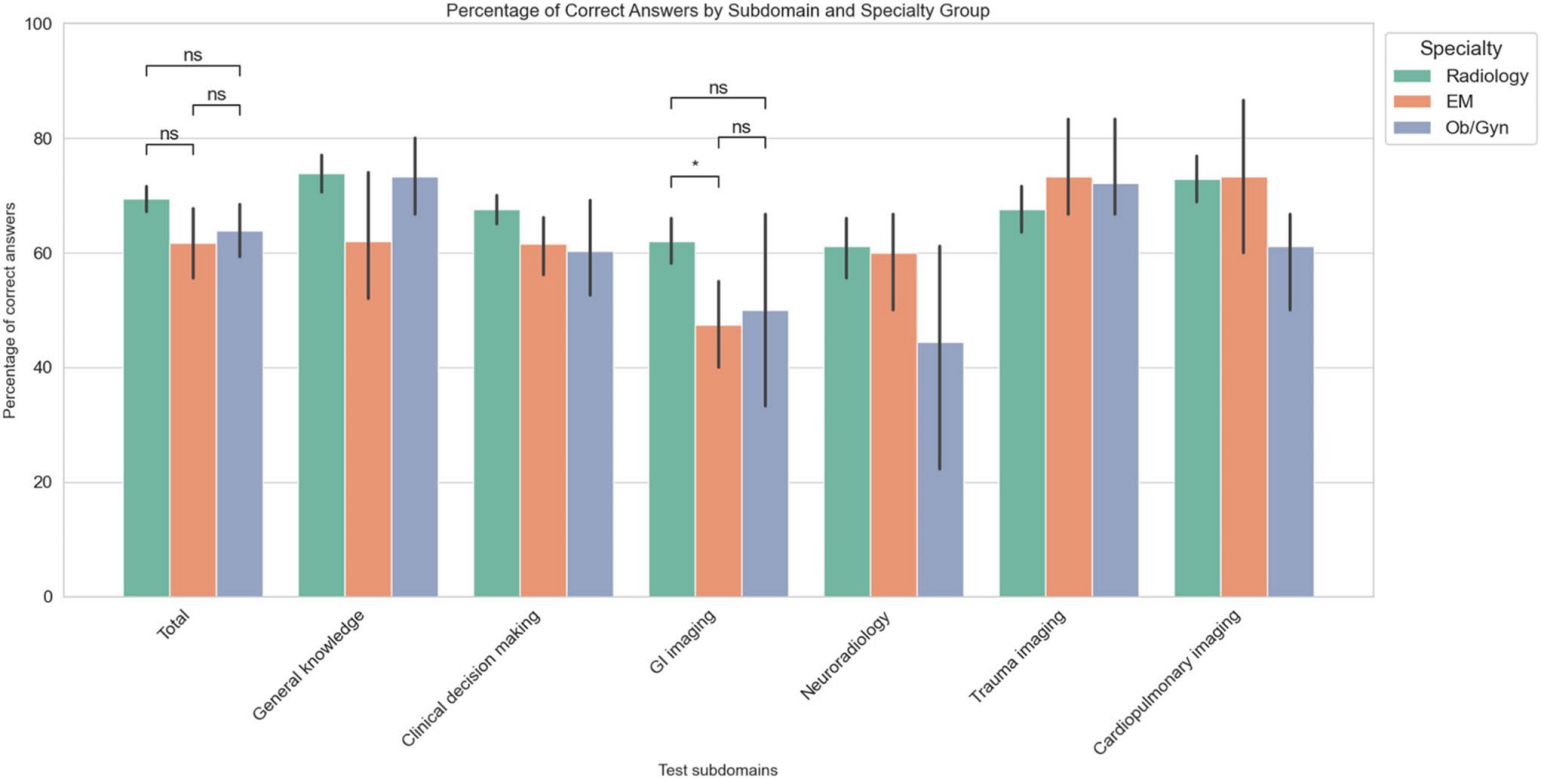
Bar plots representing the percentage of correct answers in each test subdomain, stratified by respondent specialty, along with error bars representing 95% confidence intervals and results of the post-hoc analysis in subdomains with significant findings. EM: Emergency radiology; GI: Gastrointestinal; ns: non-significant; OB/GYN: Obstetrics and gynecology

Table 5 And Fig. 5 detail correct response prevalence by specialty. Significant differences in overall scores and GI imaging scenarios were found according to specialty (*p* = 0.049 and *p* = 0.012, respectively). Additionally, post-hoc analysis with FDR correction also revealed a significantly higher GI scenario score among radiologists compared to EM physicians (*q* = 0.02) but no pair-wise between group difference was significant after FDR correction in total scores (Fig. 5). Additionally, between various radiology subspecialties, there were no significant differences in overall test performance or within question categories or subcategories (Table 6)

**Table 5:** Test scores, stratified by specialty

Specialty							
Score (Percentage of correct answers)	Overall, *N* = 124^a^	EM, *N* = 10^a^	Ob/Gyn, *N* = 6^a^	Radiology, *N* = 108^a^	*p*-value^b^	Post-hoc *p*-value^c^	Post-hoc q-value^d^
**Total score** **(% of 18 questions)**	69.4% [61.1 − 77.8%]	61.1% [55.6 − 70.8%]	61.1% [61.1 − 69.4%]	72.2% [61.1 − 77.8%]	**0.049**	Radiology vs. Ob/Gyn: 0.151 **Radiology vs. EM: 0.037** EM vs. Ob/Gyn: 0.867	Radiology vs. Ob/Gyn: 0.226 Radiology vs. EM: 0.111 EM vs. Ob/Gyn: 0.867
**General knowledge** **(% of 5 questions)**	80.0% [60.0 − 80.0%]	60.0% [45.0 − 75.0%]	80.0% [65.0 − 80.0%]	80.0% [60.0 − 80.0%]	0.14	NA	NA
**Clinical decision making** **(% of 13 questions)**	69.2% [59.6 − 76.9%]	61.5% [55.8 − 69.2%]	57.7% [53.8 − 67.3%]	69.2% [61.5 − 76.9%]	0.059	NA	NA
**GI imaging** **(% of 4 questions)**	75.0% [50.0 − 75.0%]	50.0% [50.0% − 50.0%]	50.0% [31.3 − 68.8%]	75.0% [50.0 − 75.0%]	**0.012**	Radiology vs. Ob/Gyn: 0.137 **Radiology vs. EM: 0.007** EM vs. Ob/Gyn: 0.608	Radiology vs. Ob/Gyn: 0.207 **Radiology vs. EM: 0.022** EM vs. Ob/Gyn: 0.608
**Trauma imaging** **(% of 3 questions)**	66.7% [66.7% − 66.7%]	66.7% [66.7% − 66.7%]	66.7% [66.7% − 66.7%]	66.7% [66.7% − 66.7%]	0.6	NA	NA
**Neuroradiology** **(% of 3 questions)**	66.7% [33.3 − 66.7%]	66.7% [66.7% − 66.7%]	50.0% [33.3 − 66.7%]	66.7% [33.3 − 66.7%]	0.3	NA	NA
**Cardiopulmonary imaging** **(% of 3 questions)**	66.7% [66.7 − 100.0%]	66.7% [66.7 − 91.7%]	66.7% [66.7% − 66.7%]	66.7% [66.7 − 100.0%]	0.3	NA	NA

a: Median score% [25 − 75%]

b: Kruskal-Wallis rank sum test

c: Dunn’s post-hoc test

d: False Discovery Rate corrected

EM: Emergency medicine; GI: Gastrointestinal; IQR: Inter-quartile range; Ob/Gyn: Obstetrics and gynecology

D-RAD: Diagnostic radiology; E-RAD: Emergency radiology; GI: Gastrointestinal; IQR: Inter-quartile range; I-RAD: Interventional radiology; N-RAD: Neuroradiology

**Table 6:** Test scores, stratified by sub-specialty among radiologists

			Radiology Subspecialties			
Score (Percentage of correct answers)	Overall, *N* = 108^a^	D-RAD, *N* = 64^a^	E-RAD, *N* = 32^a^	I-RAD, *N* = 6^a^	N-RAD, *N* = 6^a^	*p*-value^b^
Total score (% of 18 questions)	72.2% [61.1 − 77.8%]	66.7% [61.1 − 77.8%]	77.8% [66.7 − 79.2%]	72.2% [58.3 − 77.8%]	72.2% [58.3 − 77.8%]	0.2
General knowledge (% of 5 questions)	80.0% [60.0 − 80.0%]	80.0% [60.0 − 80.0%]	80.0% [60.0 − 80.0%]	60.0% [45.0 − 75.0%]	70.0% [60.0 − 80.0%]	0.4
Clinical decision making (% of 13 questions)	69.2% [61.5 − 76.9%]	69.2% [53.8 − 76.9%]	76.9% [67.3 − 76.9%]	73.1% [63.5 − 76.9%]	73.1% [63.5 − 76.9%]	0.2
GI imaging (% of 4 questions)	75.0% [50.0 − 75.0%]	75.0% [50.0 − 75.0%]	75.0% [50.0 − 75.0%]	62.5% [50.0 − 75.0%]	62.5% [31.3 − 75.0%]	0.6
Trauma imaging (% of 3 questions)	66.7% [66.7% − 66.7%]	66.7% [58.3 − 66.7%]	66.7% [66.7% − 66.7%]	66.7% [66.7 − 91.7%]	83.3% [66.7 − 100.0%]	0.2
Neuroradiology (% of 3 questions)	66.7% [33.3 − 66.7%]	66.7% [33.3 − 66.7%]	66.7% [66.7 − 100.0%]	66.7% [66.7% − 66.7%]	50.0% [33.3 − 66.7%]	0.058
Cardiopulmonary imaging (% of 3 questions)	66.7% [66.7 − 100.0%]	66.7% [66.7 − 100.0%]	66.7% [66.7 − 100.0%]	66.7% [66.7 − 91.7%]	66.7% [66.7% − 66.7%]	0.7

a: Median% [25 − 75%]

b: Kruskal-Wallis rank sum test

D-RAD: Diagnostic radiology; E-RAD: Emergency radiology; GI: Gastrointestinal; IQR: Inter-quartile range; I-RAD: Interventional radiology; N-RAD: Neuroradiology

(*: q < 0.05; FDR corrected)

### Test scores according to training level

Table 7 offers a breakdown by training level, with significant differences in the GI imaging subdomain (*p* = 0.03). Additional post-hoc analysis with FDR correction confirmed significantly lower correct response rates among resident physicians compared to attending physicians in the GI imaging subdomain (*q* = 0.03).

**Table 7:** Test scores, stratified by training level

		Specialty					
Score (Percentage of correct answers)	Overall, *N* = 124^a^	**Attending**, *N* = 109^a^	Fellowship, *N* = 3^a^	Residency, *N* = 12^a^	*p*-value^b^	Post-hoc *p*-value^c^	Post-hoc q-value^d^
Total score (% of 18 questions)	69.4% [61.1 − 77.8%]	72.2% [61.1 − 77.8%]	72.2% [66.7 − 72.2%]	61.1% [61.1 − 68.1%]	0.4	NA	NA
General knowledge (% of 5 questions)	80.0% [60.0 − 80.0%]	80.0% [60.0 − 80.0%]	60.0% [50.0 − 70.0%]	80.0% [60.0 − 80.0%]	0.5	NA	NA
Clinical decision making (% of 13 questions)	69.2% [59.6 − 76.9%]	69.2% [61.5 − 76.9%]	69.2% [69.2 − 73.1%]	61.5% [53.8 − 69.2%]	0.2	NA	NA
GI imaging (% of 4 questions)	75.0% [50.0 − 75.0%]	75.0% [50.0 − 75.0%]	75.0% [62.5 − 75.0%]	50.0% [43.8 − 50.0%]	**0.030**	**Residency vs. Attending: 0.009** Residency vs. Fellowship: 0.117 Attending vs. Fellowship: 0.707	**Residency vs. Attending: 0.028** Residency vs. Fellowship: 0.176 Attending vs. Fellowship: 0.707
Trauma imaging (% of 3 questions)	66.7% [66.7% − 66.7%]	66.7% [66.7% − 66.7%]	100.0% [83.3 − 100.0%]	66.7% [66.7 − 75.0%]	0.2	NA	NA
Neuroradiology (% of 3 questions)	66.7% [33.3 − 66.7%]	66.7% [33.3 − 66.7%]	33.3% [33.3 − 50.0%]	66.7% [58.3 − 66.7%]	0.4	NA	NA
Cardiopulmonary imaging (% of 3 questions)	66.7% [66.7 − 100.0%]	66.7% [66.7 − 100.0%]	66.7% [66.7% − 66.7%]	66.7% [66.7% − 66.7%]	0.8	NA	NA

a: Median% [25%% − 75%%]

b: Kruskal-Wallis rank sum test

c: Dunn’s post-hoc test

d: False Discovery Rate corrected

GI: Gastrointestinal; IQR: Inter-quartile range

## Discussion

Throughout this study, we assessed general and clinical knowledge among radiologists, OB/GYN, and EM clinicians regarding best practices in the emergency imaging of pregnant patients. In addition, we hoped to evaluate and improve physicians’ knowledge of the current guidelines surrounding imaging during pregnancy to ensure that patient care isn’t compromised due to fear of radiation.

While on certain questions, performance was strong with over 90% correct answers (#4, #5, #11–13), our study revealed lapses in other domains with less than 50% correct answers on four questions within the questionnaire (#7, 9, 15, and 18). Question #7 of our survey discusses how the radiation exposure for both mother and fetus differs between the following tests: CT pulmonary angiogram (CTPA) and ventilation/perfusion (V/Q) scintigraphy, assuming no dose-modulation techniques were utilized. Current data indicates that the mean maternal effective dose ranges from 0.23 to 9.7 milliSievert (mSv) with CTPA and 0.9 to 5.85 mSv with V/Q lung scanning. The fetal absorbed dose ranges from 0.002 to 0.51 milliGray (mGy) with CTPA and 0.2 to 0.7 mGy with V/Q scintigraphy [17]. In our survey, only 33% of respondents correctly answered this question. Other studies have reported knowledge gaps on this topic as well. In a survey by Groves et al., the authors reported that only 60% of radiologists and a little over half of other clinicians knew that V/Q scintigraphy yields a higher radiation dose to the fetus than CTPA [18]. However, in our survey, some respondents may not have realized the question was framed without dose-modulation techniques. ACR guidelines state that while the radiation dose to the fetus is lower with V/Q scanning, using dose-modulation techniques with CT may make the absorbed dose between the two modalities nearly equivalent [19], and this answer was the second most popular choice, with 29% of responses. Low-dose perfusion-only scanning has also been shown to be similarly efficacious to CTPA while decreasing radiation exposure for the maternal breast, whole body, and fetus compared to CTPA and V/Q [19–22]. Its similarity to V/Q scanning and its absence amongst the answer choices despite its viability as an alternative may have created confusion, contributing to a low rate of correct responses. These statistics ultimately highlight the fact that educational methodology regarding dosimetry necessitates further review. Furthermore, continual optimization and effective dissemination of protocols are necessary as such advancements occur.

Another question that less than 50% of respondents answered correctly was #18, which asked what imaging techniques could be used to localize a subarachnoid hemorrhage in a pregnant patient. However, only 41.9% of respondents chose magnetic resonance angiography (MRA), one of the correct answers. It is important to note that this discrepancy may have occurred due to the phrasing of the answer choice. MRA may have been interpreted as contrast-enhanced MRA, a test not often utilized in pregnant patients. Perhaps if the answer choice had been written as a non-contrast enhanced MRA (Time-of-flight /phase contrast MRA), clinicians would’ve more readily chosen this answer choice.

Regarding imaging following blunt trauma, for question #8 of the survey, 79% (98/124) of respondents correctly answered that an abdominal/pelvic CT scan was most appropriate for a 34-week pregnant patient after a negative Focused Assessment with Sonography in Trauma (FAST) exam following a motor vehicle accident. Hansen et al. reported similar findings in their study with 88% of radiologists choosing CT as the preferred modality for imaging of a 30-week pregnant patient following an inconclusive initial ultrasound for a patient with abdominal trauma in a motor vehicle accident [23].

In our analysis by training level, significant differences were detected regarding the primary knowledge sources utilized by clinicians. Medical residents predominantly consulted attending physicians/colleagues (7/12, 58%), whereas it was far less common for attendings to do so (15/109, 14%). These results were anticipated based on the fact that trainees are supposed to ask their superiors by design of the medical system. Our study additionally highlights the need for increased clinician and radiologist awareness of established societal practice guidelines to help support clinical decision-making. Just 48% (60/124) of those surveyed drew upon the ACR guidelines as a primary reference source, with utilization lower amongst OB/GYN and EM physicians compared to radiologists. However, we did not directly assess the relationship between knowledge sources and knowledge, and further study in this domain is warranted to develop and target interventions to improve physician awareness of available reference resources.

As far as confidence, most physicians expressed being either “fairly” (58/124, 47%) or “very” (51/124, 41%) confident in making imaging decisions about pregnant patients. Prior reports have also shown that physicians, including trainees, are often imprecise in assigning confidence ratings that align with diagnostic accuracy [24]. In a study by Meyer et al., internal medicine physicians correctly diagnosed 55.3% of uncomplicated cases and just 5.8% of more challenging cases, yet the associated confidence levels were relatively similar (7.2 vs. 6.4 out of 10, respectively) [24]. Further, these authors noted that higher confidence was related to decreased requests for additional diagnostic tests (*p* = 0.01) [24]. Overconfidence as such may impede clinicians from drawing upon the necessary resources to promote effective clinical decision-making.

One strength of our analysis was the anonymity, which reduces the risk of social desirability bias of answers. Our survey was also distributed internationally, which strengthens the generalizability. Yet, most participants were from the United States, and thus, our results are most applicable to this group. The strength of conclusions drawn from the international participants remains limited by the small sample size. Out of the participants who reported their country of residence, just 14.7% (15/102) were from countries other than the United States. These participants were also spread diffusely amongst such countries, with just 6.9% (7/102) from Canada, 3.9% (4/102) from Hungary, and 1.0% (1/102) from India, Italy, Saudi Arabia, and Slovenia each. In the present study, we did not collect information on the practice setting nor resources available to radiologists. Clinicians in various countries may differ significantly in the resources available to them and the protocols and care team structures, which may have limited the validity of the survey for certain respondents’ practice scenarios. For example, one report from Gujarat state in India found that X-ray and CT services availability was rarely fully adequate due to both the absence and shortage of hardware as well as staffing shortages [25]. These issues were further exacerbated by prolonged equipment breakdowns due to a lack of technicians and engineers [25].

While prior studies on imaging knowledge and practice focused on attending radiologists alone [23], our sample also included OB/GYN and EM physicians and trainees. However, while 108 respondents were radiologists, only 10 EM and 6 OB/GYN physicians participated. Such a sample may have skewed certain results in analyses with stratification by specialty, such as regarding knowledge sources consulted in decision-making, where the ACR guidelines were the most frequently consulted (60/124, 48%). For example, the ACR guidelines were most popular amongst radiologists, the largest group in our sample. EM physicians consulted it as a primary resource less often than radiologists, and OB/GYN clinicians reported not using it as a primary source at all. It is important to note that OB/GYN and EM physicians more likely consult the ACOG or the American College of Emergency Physicians guidelines, which likely explains this disparity. Further, the small sample of OB/GYN and EM physicians limits the strength of our conclusions, such as those noting differences between specialties in decision-making frequency and in general and GI subdomain test results. Similarly, our sample consisted of primarily attending physicians (*n* = 109), with just three respondents in fellowship and 12 in residency, which impacted our statistical analyses among subgroups. The survey was also lengthy, with a total of 18 questions, and even though 223 clinicians responded, we only had 124 complete responses. Lastly, the physicians who opted to participate in the survey may differ characteristically from those who did not, leading to potential non-response bias.

## Conclusion

While prior studies have focused on radiologist education and imaging practices, this is the first study that evaluated the knowledge base of multi-disciplinary clinicians regarding imaging pregnant patients in the emergent setting. Our study highlights several knowledge gaps among physicians of various specialties involved in clinical decision-making with respect to imaging of pregnant patients. While concerns regarding harm to the mother and fetus often make decisions difficult for clinicians, the risk is typically much lower than expected, and these modalities should not be withheld if clinically indicated. Though guidelines regarding this matter currently exist [5, 11], clinicians are not always aware of them. Future research aimed at evaluating and improving the dissemination of guidelines and clinician education may aid in enhancing best practices and patient outcomes in the emergent setting. Ultimately, this study highlights the need for improved education and awareness to help clinicians make informed decisions when imaging pregnant patients in the emergent setting.

## Data Availability

The survey data and results in Table 1 and Figures 1-5 can be made available upon request.
