# Correction to: Emergency imaging protocols for pregnant patients: a multiinstitutional and multi- specialty comparison of physician education

**DOI:** 10.1007/s10140-024-02290-6

**Published:** 2024-11-13

**Authors:** Liesl Eibschutz, Max Yang Lu, Payam Jannatdoust, Angela C. Judd, Claire A. Justin, Brandon K.K. Fields, Natalie L. Demirjian, Madan Rehani, Sravanthi Reddy, Ali Gholamrezanezhad

**Affiliations:** 1 Department of Internal Medicine, University of Virginia Hospital, Charlottesville, VA USA; 2 Division of Emergency Radiology, Los Angeles General Hospital, 90033 Los Angeles, CA USA; 3 Tehran University of Medical Sciences, Tehran, Iran; 4 Department of Obstetrics and Gynecology, Icahn School of Medicine at Mount Sinai/South Nassau, New York, NY USA; 5 Department of Pediatrics, Stanford University, Palo Alto, CA USA; 6 Department of Radiology & Biomedical Imaging, University of California, San Francisco, San Francisco, CA USA; 7 College of Medicine – Tucson, University of Arizona, Tucson, AZ USA; 8 Department of Radiology, Massachusetts General Hospital, Cambridge, MA USA; 9 Department of Medicine, Yale School of Medicine, New Haven, CT USA

**Correction to: Emergency Radiology (2024)**

10.1007/s10140-024-02284-4

The original version of the article contains error. There is a typo error in the first sentence of the Abstract section. It should read “providers perceive the teratogenic risks of radiologic imaging to be higher” instead of “providers perceive maging to be higher”.

The original article has been corrected.

